# Predicting infectious complications in neutropenic children and young people with cancer (IPD protocol)

**DOI:** 10.1186/2046-4053-1-8

**Published:** 2012-02-09

**Authors:** Robert S Phillips, Alex J Sutton, Richard D Riley, Julia C Chisholm, Susan V Picton, Lesley A Stewart

**Affiliations:** 1 Centre for Reviews and Dissemination, Alcuin College, University of York, York, YO10 5DD, UK; 2 Department of Health Sciences, 22-28 Princess Road West, University of Leicester, Leicester, LE1 6TP, UK; 3 Department of Public Health, Epidemiology & Biostatistics, The Public Health Building, University of Birmingham, Birmingham, B15 2TT, UK; 4 Department of Paediatric Oncology, Royal Marsden NHS Foundation Trust, Sutton, Surrey, SM2 5PT, UK; 5 Regional Department of Paediatric Haematology/Oncology, Leeds Teaching Hospitals Trust, Leeds, LS1 3EX, UK

**Keywords:** individual participant data meta-analysis, predictive modelling, paediatric oncology, febrile neutropenia, collaborative studies

## Abstract

**Background:**

A common and potentially life-threatening complication of the treatment of childhood cancer is infection, which frequently presents as fever with neutropenia. The standard management of such episodes is the extensive use of intravenous antibiotics, and though it produces excellent survival rates of over 95%, it greatly inconveniences the three-fourths of patients who do not require such aggressive treatment. There have been a number of studies which have aimed to develop risk prediction models to stratify treatment. Individual participant data (IPD) meta-analysis in therapeutic studies has been developed to improve the precision and reliability of answers to questions of treatment effect and recently have been suggested to be used to answer questions regarding prognosis and diagnosis to gain greater power from the frequently small individual studies.

**Design:**

In the IPD protocol, we will collect and synthesise IPD from multiple studies and examine the outcomes of episodes of febrile neutropenia as a consequence of their treatment for malignant disease. We will develop and evaluate a risk stratification model using hierarchical regression models to stratify patients by their risk of experiencing adverse outcomes during an episode. We will also explore specific practical and methodological issues regarding adaptation of established techniques of IPD meta-analysis of interventions for use in synthesising evidence derived from IPD from multiple studies for use in predictive modelling contexts.

**Discussion:**

Our aim in using this model is to define a group of individuals at low risk for febrile neutropenia who might be treated with reduced intensity or duration of antibiotic therapy and so reduce the inconvenience and cost of these episodes, as well as to define a group of patients at very high risk of complications who could be subject to more intensive therapies. The project will also help develop methods of IPD predictive modelling for use in future studies of risk prediction.

## Background

Children undergoing treatment for malignancy have an excellent chance of survival, with overall rates approaching 75% [1]. In most cases, children who die following treatment for cancer do so as a result of their disease, but despite huge improvements in supportive care, around 16% of deaths within 5 years of diagnosis are due to the complications of therapy [2,3]. One such life-threatening complication in immunocompromised children remains infection, which frequently manifests as the occurrence of fever with neutropenia [4].

In adopting a policy of aggressive inpatient intravenous antibiotic use in such episodes, the mortality rate related to these episodes has improved dramatically (from 30% in the 1970s to 1% in the late 1990s) [4]. Intensive care management is required in less than 5% of cases [5-7], although a substantial proportion of children have complications which require specialised care [7]. There remain many episodes of febrile neutropenia (FNP), possibly two-thirds or more, among patients in whom no significant infection is identified and in whom this aggressive management strategy is likely to be excessive [7].

To better inform the clinical management of children with cancer and FNP, there is increasing interest in using risk prediction models (also known as 'prognostic models') and clinical decision rules (CDRs) [8-10]. Risk prediction models utilise multiple prognostic factors in combination to predict the risk of a future health outcome for an individual on the basis of their set of prognostic factor values. A CDR recommends a particular clinical action (or inaction) for an individual on the basis of the prediction (for example, the predicted probability, or 'risk score') derived from the model.

A robust risk prediction model which identifies those children at very low risk of having a significant infection could result in reduced intensity and/or duration of antibiotic therapy in the hospital. It could also form the basis of a randomised controlled trial (RCT) of alternative management approaches (for example, ambulatory oral antibiotics vs inpatient intravenous antibiotics) and would be the ideal way of informing the sample size required by reliably predicting the proportion of events expected in a low-risk group. This would lead to reduced costs for the healthcare system and the patient and family [11], as well as potentially a better quality of life for all affected. At present, there are many differing policies for the management of FNP in practice [12,13] but a lack of agreement about how and which CDRs, if any, are used.

Assessment of the risk of adverse outcome of each episode of FNP has been undertaken by many different groups, with many of them creating a CDR which aims to allow clinicians to accurately judge risk and treat patients appropriately. However, none of these analyses have resulted in a widely used risk stratification model, and current practice is variable, both in the United Kingdom [12] and internationally [13-15]. Some centres use a risk-stratified, reduced-intensity approach, and others treat all children aggressively. The essential problems with research in this area are common across much of paediatric practice: those of rare conditions with small numbers of cases and limited collaboration in primary studies. The modelling studies that have been done have incorporated different clinical features and outcomes and have used different methodologies, and it is therefore difficult to draw meaningful conclusions from this body of evidence. Calls for collaborative trials [16-18] have led to little progress.

This setting provides an ideal opportunity to undertake a collaborative, pooled analysis of the existing data sets in the form of an individual participant data (IPD) meta-analysis. In this effort, we will collect and reanalyse the original study data, which will permit reanalysis of the same clinical features across studies using a consistent approach and provide sufficient numbers to draw more robust and reliable conclusions. The findings of this work should therefore more robustly inform practice and future therapeutic RCTs. The analysis can be approached from three methodological directions: testing existing CDRs for their ability to 'diagnose' adverse outcomes, assessing the added value of individual prognostic factors and building a more accurate predictive rule containing a parsimonious set of prognostic factors.

### Systematic reviews of existing knowledge

In preparation for this project, two systematic reviews were undertaken to assess the prior knowledge of the discriminatory ability of CDRs [19] and inflammatory serum markers (R Phillips, R Wade, T Lehrnbecher, LS Stewart, A Sutton) in children and young people with FNP. The systematic review of CDRs initially identified 2,057 potential studies and finally included 24, of which 21 had data in a usable format. It showed that two groups of studies have been undertaken to risk-stratify children who present with FNP. Researchers in the first group of studies examined the use of clinical examinations to predict radiographic pneumonia (4 studies) [20], and investigators in the second group examined more general infectious complications (20 studies) [19].

Among the studies in which general infectious complications were examined, 16 separate models were produced and contained 9 data sets used to validate previously derived models. The researchers studied a variety of outcomes with individual differences in definitions, but covered five main categories: death, critical care requirement, serious medical complications, significant bacterial infections and bacteraemia.

Only one rule could reasonably be assessed across multiple data sets: that of absolute monocyte count and temperature criteria proposed by Rackoff *et al. *[21] to exclude bacteraemia. The most appropriate meta-analysis of the rule's effectiveness led to estimates of moderate discriminatory ability, with the average probability of bacteraemia in the groups being low risk = 6% (95% CrI = 1% to 34%), middle-level risk = 18% (CrI = 3% to 37%) and high risk = 49% (95% CrI = 6 to 84%).

Of the other rules, the model of Santolaya *et al. *[22] showed a good ability to differentiate between low- and high-risk groups when a wider definition of 'serious infection' was used, with average predictive ability estimated as low risk = 13% (95% CI = 9% to 18%) and high risk = 72% (95% CI = 68% to 75%). The rule has been developed and tested in Chile and may be of limited applicability in Western Europe and North America [23]. Other rules show promise and have clinical physiological similarities, but have not undergone extensive testing.

The systematic review of the predictive value of serum markers of inflammation and infection in children presenting with FNP included 27 studies reporting over 13 different markers derived from an initial screen of 375 studies. The studies included had similar methodological challenges as well as problems with reporting and analysis. Many failed to assess whether the marker had any supplementary value over and above the simple admission data collected by the clinicians at every encounter: age, malignancy, temperature, age-corrected vital statistics and blood count.

To interpret the information on serum markers in a clinically meaningful way, we had to allow for the marked heterogeneity of the results. The quantitative pooling and a qualitative summary of the results suggested that procalcitonin might be a better discriminatory marker than C-reactive protein (CRP) and that IL-6 had a very good ability to predict documented infection. Overall the findings were uncertain and unstable, and only small amounts of new data may alter them substantially. Data for the other markers were too sparse to reasonably be interpreted, although IL-8 had significant potential value.

These reviews have a wide range of rules for the prediction of poor outcomes during episodes of FNP in children and the use of a variety of individual serum markers to predict outcome. None of the rules found has yet been subjected to the extensive geographical and temporal discriminatory validity assessments that mark the highest quality CDR. Many potential difficulties with different outcomes, variable selection and model-building have been identified. The data on serum markers were extremely heterogeneous, and only tentative conclusions could be drawn.

The problems identified are inherent in the attempt to undertake meta-analyses of aggregate data. The limitations of the reporting in published studies mean we do not have access to the exact data distribution or the full range of univariable estimates of predictive power. These issues could be addressed by attempting to collect more detailed summary data from the authors of the original studies, but this would not allow cross-study validation of different rules or attempts at alternative rule-building. To meet these challenges and to maximise the value of the information already collected by these groups and in other cohorts of children with FNP, an IPD meta-analysis will enable us to develop and test new and existing prediction models. This will provide a firmer basis for stratified treatment trials in this common and occasionally fatal complication of therapy.

### Rationale for an individual participant data analysis

Individual patient data pooled analysis in therapeutic studies have been developed for two decades to improve the precision and reliability of answers to questions regarding treatment [24,25]. It has more recently been promoted for the synthesis of diagnostic data [26] and prognostic information [27] to improve the quality of answers to important prognostic questions [28] and matters of diagnostic accuracy [29]. These techniques have been applied to real-world clinical data sets [30,31], in which they have clarified existing understanding of particular prognostic variables and enhanced understanding of how different diagnostic tests can be used [32].

The key benefits of prognostic IPD analysis generally can be summarised as follows: (1) Analyses are not restricted to those of the published results or subgroups; (2) analytical techniques, inclusion criteria and outcome definitions can be standardised across studies; (3) larger numbers of data points allow more powerful statistical conclusions to be drawn, including checking modelling assumptions and accounting for missing data at the individual level; (4) IPD can model data more appropriately, such as by analysing continuous variables on continuous scales (unlike in many prognostic studies in which data are reported as categorical variables); (5) analysis can account for clustering (for example, of patients within studies) and correlated information (for example, multiple events per individual); (6) multivariate models can be created across different healthcare settings; (7) data can be reviewed for completeness and accuracy; and (8) the analysis can provide extensive internal cross-validation to guard against data-driven exaggerations of predictive power.

In the Predicting Infectious Complications of Neutropenic sepsis In Children with Cancer (PICNICC) study, the collection and analysis of IPD will provide specific benefits that overcome many of the problems found in the aggregate data meta-analysis. Many of the benefits of IPD analysis are technical, being related to the statistical methods underlying the meta-analysis and the building of predictive models. Although at first sight the failure to address the problems inherent in statistical interpretation may seem to be clinically irrelevant, it has clear and real clinical implications [33]. Other benefits are more obviously clinical; for example, the collection of the different data sets will enable us to clarify and harmonise the different outcomes collected.

One of the primary 'statistical' benefits will be the use of firmly prespecified potential predictor variables built upon the experience of the PICNICC Collaborative and the systematic reviews. This will guard against the development of purely data-driven analyses, which have a tendency to overestimate any predictive value [28].

In the reviews, we found the studies designed to build a CDR used a large number of variables (median = 13, range = 2 to 39) and had a small number of events (median = 36, range = 4 to 178) with 70% (12 of 16) studies having fewer than 10 events per variable under consideration and no study having more than 14 events per variable. These low event-per-variable ratios render predictive conclusions drawn from the studies unstable and estimates of predictive power overly optimistic [34]. IPD will allow us to consolidate the information and increase greatly the number of events studied from the same number of predictive variables.

The raw data will also allow a detailed analysis of the clustering of events (multiple episodes per patient) and variation at the level of the individual patient. This issue is significant when assessing the problems identified in the aggregate data reviews. Multiple episodes in individual patients were treated primarily as if they came from dissimilar individuals in the 20 CDR and 24 serum marker studies. Four papers explicitly described no adjustment [22,35-37], with 12 undertaking some attempt at assessment. Secondary analysis was performed to assess 'first included case' versus 'all episodes' and 'no significant differences' in three studies [22,38,39] and in nine others in which more advanced statistical modelling was used [6,21,40-46]. In 28 studies, the assessment was unclear.

The functional form of the data regarding *a priori *nonlinear fractional polynomial relationships can be assessed in detail. In no study assessed were clear attempts made to fit nonlinear forms to the data. This is unsurprising, as the development of practical techniques to undertake this effort is very recent [47].

Modern statistical developments in the handling of missing data may enhance the information already acquired. Again, very little information on the assessment and management of missing data was available from the reviews (five CDR studies [21,38,39,42,44] and two serum marker studies [48,49]). Very recent publications of studies in which simulation [50] and surveying practice [51] were used produced workable guidelines for the use of imputation techniques to maximise the value of the data collected. IPD will allow us not only to test existing rules and combine data derived from attempts to examine the rules but also, potentially, to develop a more robust rule for future use worldwide.

### Parent and/or caregiver involvement

The development of shared research initiatives involving patients, clinicians and researchers has been a notable change in the practice of clinical research over the past decade [52]. It remains surprising to many researchers, clinicians and patients when they learn that their views are often strikingly different from each others' [53]. A systematic review of studies of the process of research planning and priority setting undertaken by the James Lind Alliance [52] demonstrated that the involvement of patients and parents as well as other caregivers was extremely infrequent.

The PICNICC group has sought to involve parents early in the treatment process. Discussions of the nature of their engagement in the process have so far highlighted that the representatives involved have not wished to be actively involved in the process of reviewing, but to be included in discussions about the nature of, the adverse effects of FNP and that they have been willing to provide their own nonmedical expertise in advancing the project.

The discussion of the nature and extent of patient and caregiver involvement in the PICNICC group will continue as the project develops. Possible opportunities for further involvement include writing commentaries on the study for patients and their families, providing alterative views on ethical questions, making choices regarding risk thresholds and considering how uncertainty and imprecision should be managed.

## Methods

### Aims

#### Primary

A primary aim of the project is to undertake an IPD pooled analysis to quantify the risk of adverse clinical outcomes according to clinical variables in children and young people undergoing treatment for malignant disease who present with an episode of FNP; that is, to identify which variables are prognostic and which have the most independent prognostic importance. Another primary aim is to develop and validate a new risk prediction model containing multiple prognostic factors in combination.

#### Secondary

The secondary aim of this project is to develop and explore practical and methodological issues surrounding the use of pooled IPD analysis in the development of predictive models.

#### Inclusion and exclusion criteria

Studies will be considered for inclusion in the IPD meta-analysis if they are cohort studies of children and young people presenting with FNP and/or with either prospective or retrospective data collection, including RCT data; if they provide data for all 'essential' predictive variables in more than 50% of included episodes (see 'Core data set and variables' section); and if they provide details of two or more study-defined outcomes in more than 90% of individual episodes of FNP.

Studies will be excluded if they are case series (for example, studies of only 'Gram-negative bacteraemias') and if they did not record data on all 'essential' predictive variables or cannot provide sufficient outcome data.

Studies will be included if they focus on the collection of data from children and young people (between 0 and 24 years old). The purpose of the inclusion criterion of studies of young people up to the age of 24 years is to address a paucity of research on individuals in the 'young adult' age range [54]. Data from individual patients ages 25 years and older will be excluded from this analysis. The median age of inclusion in the 'children's' cohorts examined in our reviews was about 7 years old (ranging from 1 month to 23 years), and the 'adult' study from the Multinational Association for Supportive Care in Cancer group [55,56] has a median age of 52 years (range, 16 to 91 years old).

### Identification of potential studies

The initial identification of studies has been through extensive literature searches undertaken as part of the systematic reviews reported briefly in the Additional material at the end of the protocol (see Appendix 1 in Additional file 1 for a list of studies).

The following databases were searched by two independent reviewers to identify potential collaborators: MEDLINE, MEDLINE in-process and other nonindexed citations, Embase, Cumulative Index to Nursing and Allied Health Literature, *Cochrane Database of Systematic Reviews*, Database of Abstracts of Reviews of Effects, Health Technology Assessment Database, Cochrane Central Register of Controlled Trials, Thomson Reuters Conference Proceedings Citation Index-Science and Literatura Latinoamericana y del Caribe en Ciencias de la Salud. The reference lists of relevant systematic reviews and included articles were reviewed for further relevant studies. Published and unpublished studies were sought, and no language restrictions were applied. Non-English-language studies were translated into English. (See Appendix 2 in Additional file 2 for a sample search that we conducted.)

Further analysis of the initial literature searches will be undertaken to identify any published cohorts of FNP patients that may have been excluded from the reviews because a CDR or serum marker was not tested, yet could provide the information essential to being included in the IPD study. In addition to this, open calls for participation have been made via the International Society for Paediatric Oncology Supportive Care Group, the University of York Centre for Reviews and Dissemination website (http://www.york.ac.uk/inst/crd/projects/picnicc_patient.htm), presentations at relevant UK and international conferences, and via the Oncopedia web community of paediatric oncologists (https://www.cure4kids.org/ums/home/index.php?location=%2Fums%2Foncopedia%2F).

### Core data set and variables

This IPD meta-analysis will develop a risk stratification model to predict which children and young people have a low risk of adverse outcomes during an episode of FNP. The predictor variables and adverse outcomes sought have been based on our systematic reviews of aggregate data, in which exploratory analysis showed that age, malignant disease state, clinical assessment of circulatory and respiratory compromise, higher body temperatures and bone marrow suppression had explanatory value and reflected clinical experience of the paediatric oncologists.

The following predictor variables are divided into 'essential' and 'desirable' items and can be categorised as (1) patient-related, episode-related clinical variables and (2) patient-related, episode-related laboratory variables:

1. Age

2. Underlying tumour type

3. Marrow involvement and/or remission status

4. Chemotherapy type and time elapsed since last cycle

5. Presence of central venous line

6. Inpatient or outpatient at onset of episode

7. Maximum temperature

8. Antibiotic therapy used

9. Respiratory rate (or compromise)

10. Circulatory parameters (or compromise)

11. Severe mucositis

12. Global assessment of illness severity

13. Haemoglobin

14. Platelet count

15. White blood cell count

16. Neutrophil count

17. Monocyte count

18. CRP

19. Procalcitonin

20. IL-6

21. IL-8

The following are outcomes of primary interest from each episode:

1. Death

2. ICU admission

3. Need for moderate organ support (fluid bolus, oxygen)

4. Clinically documented infections

5. Microbiologically documented infections

Two or more of these outcome measures should be provided for more than 90% of episodes.

If available, we will also collect data on the following:

1. Duration of fever

2. Duration of admission

An example of the initial survey of data available from collaborators is provided in Appendix 3 in Additional file 3. An *a priori *mapping schema linking microbiological and clinical outcome variables into a unified description of 'severe' and 'nonsevere' infections has been developed to assist with unifying outcome definitions.

### Providing data

#### Anonymised deidentified data

Data sets should be anonymised (that is, have all directly identifiable material removed, such as name, address, postal code, record number). A patient identification number should be provided to facilitate communication and data queries. For the purposes of this report, the age of the patient (an indirect identifier) is essential and should be provided [57].

#### Data format

The data will be accepted by the PICNICC Collaborative in any electronic format, but ideally a 'flat' spreadsheet format (such as Microsoft Excel; Microsoft Corp, Redmond, WA, USA) will be most useful, with one episode per row and variables listed in columns. Each patient should be assigned an in-cohort unique identifier (such as a simple number 1, 2 ... *n*) to highlight repeated episodes in the same patient. A suggestion for coding the variables is provided in Appendix 4 in Additional file 4 and a sample flat file is available on request.

#### Transfer of data

The data should be transferred to a secure password-protected web server or by pretty good privacy-encrypted email. This permits a secure and identifiable connection to the University of York servers and minimises the possibility of data loss.

#### Data checking

Simple checks of data integrity will be undertaken prior to analysis. These checks will include sense checking of data (for example, impossibly low presenting temperatures, such as less than 30°C or for second episodes of FNP where the outcome of the first was death), clarifying missing data (that is, ensuring missing data is recorded as 'missing' rather than 'zero') and calculating simple descriptive statistics of 'essential' elements to assess for 'outlier' studies (for example, age, sex, number of episodes per person). Any problems or inconsistencies flagged during these procedures will be discussed with the individual responsible for each study and amended as appropriate by consensus.

### Ethical and regulatory considerations

This IPD protocol has been approved in the United Kingdom by the University of York Health Services Research Ethics and Research Governance Committee. Each clinician member of the PICNICC Collaborative is advised to seek country-specific advice regarding the regulations which apply to data shared in this study.

### Plan of investigation

#### Method of analysis

The primary method of analysis for the PICNICC study will be the use of multivariable logistic regression modelling. There are a series of different analytical techniques that can be used to produce rules, including multivariable regression analysis, classification and regression tree (CART) models, discriminant analysis and neural networks. There is no clear evidence that one method is superior to any other [58], and, as multivariable logistic models have the widest clinical understanding and applicability, this method has been selected.

In the primary analysis, data used will be from the first recorded episode for each patient to predict an absence of adverse outcomes due to the individual episode (that is, death, intensive care requirement, medical complication, bacteraemia or other significant bacterial infection). Following the primary analysis, outcome data and predictor variables from subsequent episodes will be analysed to assess the independence or otherwise of these data, and this information will also be included using an appropriate model.

Prospective and retrospective cohorts will be considered separately in the initial analyses on the basis of the hypothesis that there will be a clinically important difference between the two types of studies. If no difference is found, then the data set will be examined as a whole. The prognostic importance of individual variables, both unadjusted and adjusted for other variables (the latter to summarise independent prognostic value), will be summarised for each study.

### Assessment of study and data quality

There is very little advice in the literature for assessing the quality of prognostic studies. Altman and Lyman presented suitable criteria that those initiating a primary prognostic study should consider [59], and they suggested that every effort should be made to limit potential biases and to emulate the design standards of a clinical trial. Ideally, the data should be collected prospectively, with little missing data for predictors or outcomes and with predefined hypothesises. We will use these guidelines and those published by Hayden *et al. *[60] to help inform the quality of the IPD obtained. For example, an assessment will be made of the proportion of missing data and the completeness of follow-up. The influence of any studies considered problematic (for example, those with large amounts of missing data or a great deal of incomplete follow-up) on the prediction model will then be considered, resulting in either their exclusion or in sensitivity analyses comparing model estimates when they are included or excluded.

### Model development

The model will initially incorporate the simplest predictor variables (malignant diagnosis, age, time since chemotherapy, and maximum recorded temperature) before standard additional variables (such as clinical assessments of compromise, inpatient or outpatient status, white blood cell counts or other haematological parameters) are added. Further specialist tests (for example, CRP and IL-6 levels) will be added. The type of antibiotic therapy used will always be incorporated into the model as a categorical variable. Potential sources of heterogeneity (for example, in effects of particular variables across studies) will be incorporated as random effects as appropriate. The models will be assessed for improvement in fit by using an information criterion (for example, Akaike's information criterion) with a *P*-value of < 0.15 used for inclusion. We will use a 15% rather than a 5% level, as we feel this is more conservative and will limit the chance of missing important covariates. At the stage of deciding our final model, however, we will check that the model's predictive accuracy (discriminatory ability) is improved by the inclusion of variables whose significance is between 5% and 15%. If predictive accuracy is not improved, then these variables will be removed.

This approach (of adding specialist tests only after considering the simpler tests) maximises the utility of a model by ensuring that, if extra tests with their additional costs are required, they will add considerable predictive power to existing simpler variables [61]. We will use bootstrapping and shrinkage to adjust for potential overoptimism (bias) in parametric estimates and trends.

Continuous candidate variables will be assessed using the best fitting functional form considering appropriate transformations or fractional polynomials (also assessed using an information criterion) as suggested by previous evidence. Missing data will be examined to define the nature of the 'missingness'. If they are missing at random, then multiple imputation techniques will be used to address these gaps utilising all the other available data [50,51]. The results of these analyses will be compared with a complete case analysis. We will conduct an analysis comparing the new model that we develop with other validated models, for example, that of Santolaya *et al. *[62]. This will provide an opportunity to test these CDRs against data from other geographical areas.

We acknowledge that there may be unforeseen challenges caused by the variations in the data formats available from the different studies. Therefore, we acknowledge that establishing the definitive analysis plan will be an iterative process and may even demand novel methodological developments (see 'Further research opportunities arising from PICNICC' section).

### Assessing model performance

An important goal of a prediction model is to classify patients into risk groups. The developed model will produce a risk score for each individual that is based on the patient's own predictor values. We will then use a cutoff value to decide when a risk score is high (such that we predict an adverse outcome) and when it is low (such that we predict a good outcome); this will be our CDR. The calibration of the model will be assessed by classifying children into deciles ordered by predicted risk and considering the agreement between the mean predicted risk and the observed events in each decile. The derived CDR will be cross-validated by comparing the classification of each patient with his or her actual outcome, thus allowing an estimate of the sensitivity and specificity of the prediction model. Next, by varying the chosen cutoff level, we will be able to produce a receiver operating characteristic curve (ROC) summarising the sensitivity and specificity of the predictive rule across the range of cutoffs. The overall discriminatory ability will be summarised as the area under the ROC (AUC ROC) with the 95% confidence interval. The most suitable cutoff level can then also be detected.

Each predictive model will be tested by checking how it performs against the data from all but one of the studies in turn (cross-validation of intrinsic prognostic performance) [63] and by using the bootstrap procedure [64]. This will adjust for overoptimism in the estimation of model performance due to validation in the same data set that was used to develop the model itself.

The improvement in model performance by adding prognostic factors will be assessed by net reclassification improvement. By analysing the difference among the prognostic factors, a shrinkage factor will be calculated and the model will be corrected by this shrinkage factor. Note also that clustering of patients within studies will be accounted for in the model framework.

### Validation in new data

We will compare the predicted and observed event rates to assess calibration (as described above) and the AUC ROC to assess discriminatory ability. If new data become available after the formation of the PICNICC Collaborative, they will provide an excellent test bed for the newly proposed model. Such an analysis is outside the initial scope of this project. We will update the model if it shows poor performance to adjust it to the new situation by recalibration or revision methods, depending on discrimination performance. Simple diagnostic test accuracy measures (such as positive and negative predictive values) will be computed for a hypothetical population (with its particular incidence rates) to aid clinical interpretation of the study results that define a low-risk group.

### Assessment of publication bias

We do not believe that publication bias will affect the data we obtain. We have sought to retrieve full data from the studies and so have sidestepped many of the problems of reporting bias. There may remain issues of different outcome collection and different outcome assessment methods, but these will not have been biased by collection and analysis of the predictive data. We have tried to avoid publication bias by making open calls for data which has been collected but not yet published, and we have probably secured three such data sets for analysis. This may be too few to undertake a formal assessment of the difference between the published and unpublished sources. We are also using the data for a purpose different from that used by the original data collectors. We are developing a prediction model, whereas the original researchers are interested only in the prognostic effect of particular variables. Furthermore, by obtaining the IPD, we have obtained outcomes and variables not reported by the original data collectors in any publication. However, to check whether our collection of studies may be affected by publication bias, we will display a funnel plot for each of the variables included in the final model to see whether there is asymmetry (that is, potential publication bias). We will use guidelines for assessing asymmetry recently published in *BMJ *[65].

### Publication policy

The main results of the meta-analysis will be published and presented under the PICNICC name, with PICNICC comprising groups supplying data for analysis as well as its advisory group. Any subsequent technical papers which describe innovations in the methodologies used in the meta-analysis will acknowledge the PICNICC Collaborative as the source of the data. The PICNICC Collaborative will disseminate the findings of its research widely at academic conferences and in journal publications, on the University of York website and in lay summaries of the research.

## Discussion

### Status of the project

Currently, the PICNICC Collaborative has completed study identification and invitation and has collected data derived from 23 data sets from 12 countries, including the Europe-wide European Organisation for Research and Treatment of Cancer studies. No data analyses have yet been undertaken. The opportunity to include data sets for the derivation of a new PICNICC CDR have now closed, but approaches may be made to the authors for consideration of inclusion of further data sets in subsequent validation testing or further refinements of the initiative.

### Further research opportunities arising from PICNICC

It is hoped that collaborations developed through the PICNICC project may also lead to a series of international studies to improve patients' experiences and outcomes with regard to infectious complications in cancer. One obvious follow-up study might be the use of the newly derived model in a RCT of alternative management approaches (for example, ambulatory oral antibiotics vs inpatient intravenous antibiotics). Other studies may include the investigation of genetic polymorphisms in determining the outcomes of infectious episodes; the prediction of specific infections which may require different management approaches, such as antibiotic-resistant bacteraemia; or the prediction of the risk of an episode of FNP.

The PICNICC Collaborative will provide data that will prove invaluable in the development of the methodology of IPD meta-analysis for risk prediction. This developmental work, which will be essential to developing the best possible model in PICNICC, is outside the core clinical questions set for the PICNICC Collaborative and will be undertaken as a series of linked projects. The problems to be addressed in developing the methodologies will depend on the nature of the data sets obtained. They may address issues regarding the analysis of missing data, the use of different imputation models, the modelling of multiple-episode data, the relative merits of prospective and retrospectively collected information, the use of alternative modelling techniques (such as CART, structured equation modelling, Bayesian techniques or neural networks), the comparison of episodic and patient-centred analyses and the use of categorical outcome variables. A short methodological protocol will be developed for each methodological investigation prior to commencement.

## Abbreviations

95% CI: 95% confidence interval; 95% CrI; 95% credible interval: CART: classification and regression tree; CDR: clinical decision rule; CRP: C-reactive protein; FNP: febrile neutropenia; IL: interleukin; IPD: individual participant data; RCT: randomised controlled trial.

## Competing interests

The authors declare that they have no competing interests.

## Authors' contributions

RSP conceived, developed and drafted the protocol and the systematic reviews referenced herein. He was supported in the clinical details by JCC and SVP. Statistical advice and support were provided by AJS and RDR. The project was overseen and developed with LAS, who also contributed extensively to the practical processes of undertaking an IPD meta-analysis. All authors read and approved the final manuscript.

## Supplementary Material

Additional file 1 **Appendix 1: Potential IPD Datasets**. Click here for file

Additional file 2 **Appendix 2: Search Strategy**. Click here for file

Additional file 3 **Data collection survey**. Click here for file

Additional file 4 **Suggested coding structure**. Click here for file
