# Supplementary material for: Predicting infectious complications in neutropenic children and young people with cancer (IPD protocol)
Source: Syst Rev. 2012 Feb 9;1:8. doi: 10.1186/2046-4053-1-8 (PMC3351734; doi:10.1186/2046-4053-1-8)
Supplement: Additional file 1 — Appendix 1: Potential IPD Datasets. [file 2046-4053-1-8-S1.DOCX]

# Appendix 1: Potential IPD Datasets

| **Citation** | **Type of study** | **n**  **pts** | **n epi** | **n evts** | **All candidate variables initially examined** | **Outcomes** |
| --- | --- | --- | --- | --- | --- | --- |
| Adcock 1999 | Retrospective | 33 | 88 | 16 | Demographics, primary diagnosis, history of present illness, vital signs, and physical examination. Recent chemotherapy regimen, prophylactic (antibiotic) therapy, leukocyte count and ANC, maximum daily temperature, age, and condition of central line | Gram positive bacteremia |
| Alexander 2002 | Retrospective | 104 | 104 | 13 | Anticipated neutropenia <7 days, no significant comorbidity at presentation (defined later). | Bacteraemia Serious medical complication Death |
| Ammann, 2003  (models #1 - #3) | Retrospective | 111 | 285 | 90 | 39 variables: age, gender, pre-B-cell leukaemia or other diagnosis, first or later malignancy, relapsed or unrelapsed malignancy, history of episodes of FN without significant bacterial infection, history of episodes of FN with significant bacterial infection, history of episodes of FN with bacteraemia, remission status of malignancy, bone marrow involvement, maintenance therapy or more intensive chemotherapy, delay since last chemotherapy, time since diagnosis, year of previous episode(s) of FN, season of previous episode(s) of FN, preventive application of G-CSF, central venous catheter present, hospitalisation history before FN, presence of comorbidity requiring hospitalisation, iatrogenic reason for fever, fever rule (≥38.5°C persisting for at least 2 hours or once ≥39°C), weight loss since last chemotherapy, BMI, maximal fever at presentation, general appearance, presence of chills at presentation, lowest systolic BP, lowest diastolic BP, presence of oral mucositis, presence of clinical signs of viral infection, haemoglobin level, leukocyte count, neutrophil count, monocyte count, phagocyte count, thrombocyte count, serum CRP level, serum creatinine level, and serum ASAT level. | Severe bacterial infection, (death from bacterial infection, a positive culture of normally sterile body fluids, radiologically proven pneumonia, clinically unequivocal diagnosis of a bacterial infection, or CRP>150 mg/L) |
| Ammann, 2004 | Retrospective | 132 | 364 | 85 |  |  |
| Hann 1997 | Retrospective (RCT trial data) | 759 | 759 | 165 | Gender, underlying disease (AML, ALL, BMT, HD/NHL, CML-aplasia-blast-crisis-other, Solid tumour), disease status (induction, relapse, maintenance), IV line in situ, defined site of infection, shock, granulocyte count, period of granulocytopenia, antifungal prophylaxis, antibacterial prophylaxis, age, temperature, (log) creatinine | Bacteraemia |
| Jones 1996 | Prospective | 127 | 276 | 68 | Underlying disease and status (i.e. induction therapy, remission or relapse). Age at time of fever episode. ANC at time of onset of fever. Inpatient versus outpatient status | Bacteraemia Clinical infection |
| Klaassen, 2000 | Prospective | 140 | 227 | 28 | 13 variables assessed: age, presence of bone marrow disease, central venous catheter type, general appearance on initial examination, previous granulocyte colony-stimulating factor (G-CSF) therapy, initial ANC, initial lymphocyte count, initial monocyte count, initial platelet count, presence of localized bacterial infection on initial examination, peak temperature, tumour type, sex. | Bacteraemia Significant bacterial infection (defined as any blood or urine culture positive for bacteria, interstitial or lobar consolidation on CXR, or unexpected death from infection before ANC recovery (>0.5 x 109/L)) |
| Lucas, 1996 | Retrospective | 161 | 509 | 82 | Chills, hypotension, poor perfusion, the need for fluid resuscitation, time from cytotoxic chemotherapy, diagnosis, disease status, and the presence of a focus of infection | Positive blood culture ICU Septic death |
| Paganini 2007 | Prospective | 458 | 714 | 18 | Age, days since chemotherapy, ‘advanced stage of disease’ (= bone marrow involvement, relapse, second tumour, high-dose therapy, genetic disease), previous antibiotic or CSF use, ANC <100, clinical infection, pneumonia, mucositis, bacteremia <24h, comorbidity (=incoercible bleeding, refractory hypoglycaemia and hypocalcemia, hypotension, altered mental status, renal insufficiency, hepatic dysfunction, and respiratory failure). They also state that the following variables were collected and registered for analysis: facial, anal, oral or catheter-associated cellulitis, sepsis, necrotising gingivitis, sex, underlying disease and staging, predicted period of neutropenia, presence of intravenous device. | Death |
| Rackoff, 1996 | Prospective (Derive) | 72 | 115 | 24 | State of disease (remission vs not), degree of mucositis, ill appearance, presence of GI symptoms, cellulitis, use of GCSF, admission ANC, admission AMC, maximum admission temperature | Bacteraemia Clinical reason for admission |
| Rackoff, 1996 revised model | Retrospective (Validate) | 102 (see note) | 57 | 10 | AMC, Temperature (39.5C cutoff), ANC, APC, Platelets, age, WBC | Bacteraemia Clinical reason for admission |
| Riikonen 1993 | Prospective | 46 | 91 | 17 | Duration of fever, duration of neutropenia, central line present, prophylaxis with Septrin, general clinical examination, HR, signs of bleeding, BP, temperature, chills, Hb, Plt, prolonged PTT, sodium & potassium ESR, CRP | Bacteraemia Suspected sepsis/Fever of Unknown Origin Focal infection |
| Rojo, 2008 | Retrospective | 33 | 47 | 4 | Sex, age, type of malignancy (leukaemia vs solid), focus of infection, duration of hospitalisation, microbiologically proven infection | ‘Unfavourable outcome’ - Compound of: haemodynamic instability, new focus if bacterial infection, 72h persistent fever, unresponsive CRP, or continuing +ve blood cultures 72 hours after treatment |
| Rondinelli, 2006 | Retrospective | 283 | 283 | 93 | Significantly on univariate: Age, gender, disease type (AML, ALL, Others), disease status (remission/other), CVC, temperature, Hb, WCC, AGC, Plt, AMC, URTI, time from chemotherapy, pneumonia, clinical site of infection, mucositis plus others not reported | ‘Serious infectious complication’ – sepsis, shock, +ve blood cultures, infection-related death |
| Santolaya, 2001 | Prospective | 257 | 447 | 178 | (1) demographic variables, ie, age, sex, and maternal educational level; (2) cancer-related variables, ie, cancer type, intensity of chemotherapy, use of granulocyte colony-stimulating factors since last administration of chemotherapy, and use of an indwelling catheter; (3) variables related to the febrile episode, ie, hours of fever before admission, days since last administration of chemotherapy, and use of prophylactic antimicrobial agents; (4) admission clinical and laboratory variables, ie, axillary temperature, blood pressure, ANC, AMC, quantitative serum CRP level, hemoglobin level, and platelet count | Invasive bacterial infection (positive blood culture – 2 for CoNS, positive bacterial culture from usually sterile site, or sepsis syndrome and/or focal organ involvement and haemodynamic instability and severe malaise) Death |
| Tezcan 2006 | Retrospective | 240 | 621 | 143 | Age, sex, ANC, AMC, CRP, duration of neutropenia, duration of fever, presence of previous FN, presence of hypotension, uncontrolled malignancy, cancer type. | Death Clinically suspected infection Microbiologically documented infection |
| West, 2004 | Retrospective | 143 | 303 | 36 | Age, type of cancer, chills, temperature, HR, RR, SBP, DBP, mucositis, Hb, Plts, WCC, differential WCC, ANC, AMC, monocytes <10%, perirectal abscess, capillary refill time >3s. | Requirement for critical care within 24 hours of presentation (fluid boluses ≥60ml/kg, inotropes or ventilation) |
| Barnes 2002 | Prospective | 37 | 39 | 26 | PCT | Stay of <5d or 5d |
| de Bont 1999 | Prospective | 19 | 72 | 1 | CRP, IL6, IL8, Age, sex, type of malignancy, leucocyte count | Bacteraemia |
| Diepold 2008 | Prospective | 69 | 123 | 14 | IL6, IL8, CRP | BacteraemiaOther - Fever lasting ≥5d but culture -ve |
| Dylewska 2005 a & b | Retrospective | 66 | 108 | ? | PCT, CRP | BacteraemiaOther - Clinically defined infections (UTI, neurological, GI or respiratory); microbiologically defined other infection, FUO |
| El-Maghraby 2007 | Prospective | 76 | 85 | 59 | CRP, IL8, MCP | Bacteraemia or clinically documented infection |
| Hatzistilianou 2007 | Prospective | 29 | 94 | 60 | PCT, CRP | Microbiological or clinically documented infection (excludes viral) |
| Heney 1992 | Prospective | 33 | 47 | 6 | CRP, IL6 | Bacteraemia |
| Hitoglou-Hatzi 2005 | Prospective | 67 |  | 29 | CRP, PCT, tADA | Significant bacterial infection |
| Hodge 2006 | Prospective | 31 | 31 |  | IL5, IL8, IL12, CRP | Positive blood culture |
| Katz 1992 | Prospective | 74 | 122 | 7 | CRP, Tumour type (solid vs haematological) | Clinically or bacteriologically documented infection, and Septicemia (+ve blood cultures & unwell clinical appearance) |
| Kitanovski 2006 | Prospective | 32 | 68 | 16 | CRP, PCT, IL6 | Bacteremia & clinical sepsis or clinically/microbiologically documented local infection |
| Lehrnbecher 1999 | Prospective | 56 | 121 | 8 | CRP, IL8, IL6 | FUO, clinically documented,G+, G-, or fungal infection |
| Lehrnbecher 2004 | Prospective | 146 | 311 | 18 | IL6, IL8 | Significant bacterial infection(Bacteramia, localised infection or pneumonia) |
| Riikonen 1992 | Prospective | 46 | 105 | 20 | IL1, IL6, TNF, SAA | Bacteramia, 'suspected sepsis', focal infection, no infection |
| Riikonen 1993 | Prospective | 46 | 91 | 17 | CRP | Bacteramia, 'suspected sepsis', focal infection and no bacterial infection |
| Santolaya 1994 | Prospective | 75 | 85 | 55 | CRP | Documented bacterial infection (bacteremia inc two for commensals) or serile site +ve - aka SBI.,Probably bacterial infection (cultures negative but severe medical course e.g. purulent gingivostomatitis, CXR+), Viral or FUO |
| Santolaya 2007 | Prospective | 219 | 373 | 15 | CRP | Death |
| Santolaya 2008 | Prospective | 278 | 566 | 116 | CRP, IL8, PCT, Age, ANC, AMC, Plts, Serum urea, serum glucose, LDH, | Severe sepsis (sepsis + respiratory or cardiac compromise, or + 2 other-organ compromise) not apparent during the first 24h of admission |
| Secmeer 2007 | Prospective | 49 | 60 | 25 | CRP, PCT, ESR | Bacteraemia and otherDocumented bacterial infection (microbiologically or clinically), and duration of fever. |
| Soker 2001 | Prospective | 23 | 48 | 11 | IL2-R, IL6, IL8, TNF-alpha, IL1 | Bacteraemia |
| Spasova 2005 | Prospective | 24 | 41 | 14 | CRP, IL6, IL8, IL10, Association tested for age, gender, 1st/subsequent episodes, ANC, AMC, duration of neutropenia | BacteraemiaMicrobiologically or clinically proven local infections without bacteraemia. |
| Stryjewski 2005 | Prospective | 56 |  | 16 | PCT, IL6, IL8, T(max), Plt, ANC, AMC | Sepsis (positive culture - two consecutive +ve if CoNS, fever, tachycardia, or tachypnoea) or septic shock (as above plus need for inotropes/vasopressors) |
| Santolaya 2002 | Prospective | 170 | 263 | 178 | CRP, (1) demographic variables, ie, age, sex, and maternal educational level; (2) cancer-related variables, ie, cancer type, intensity of chemotherapy, use of granulocyte colony-stimulating factors since last administration of chemotherapy, and use of an indwelling catheter; (3) variables related to the febrile episode, ie, hours of fever before admission, days since last administration of chemotherapy, and use of prophylactic antimicrobial agents; (4) admission clinical and laboratory variables, ie, axillary temperature, blood pressure, ANC, AMC, hemoglobin level, and platelet count | Sepsis not presenting in first 24h |

# 
