# Supplementary material for: Predicting infectious complications in neutropenic children and young people with cancer (IPD protocol)
Source: Syst Rev. 2012 Feb 9;1:8. doi: 10.1186/2046-4053-1-8 (PMC3351734; doi:10.1186/2046-4053-1-8)
Supplement: Additional file 2 — Appendix 2: Search Strategy. [file 2046-4053-1-8-S2.DOCX]

# Appendix 2: Search Strategy

**Example based on OVID-Medline: was adapted for other databases**

*FNP identification*

1 Neutropenia/

2 (neutropenia or neutropenic).ti,ab.

3 1 or 2

4 Fever/

5 (fever$ or febril$).ti,ab.

6 4 or 5

7 3 and 6

*Child identification*

8 adolescent/ or child/ or child, preschool/ or infant/ or infant, newborn/ or Puberty/

9 schools/ or schools, nursery/

10 (infan$ or newborn$ or new born$ or baby$ or babies or neonat$ or neonat$ or child$ or schoolchild$ or kid or kids or toddler$ or adoles$ or teen$ or boy$ or girl$ or minor$ or underage$ or under age$ or juvenil$ or youth$ or kindergar$ or nursery or puber$ or prepuber$ or pre puber$ or pubescen$ or prepubescen$ or pre pubescen$ or pediatric$ or paediatric$ or peadiatric$ or school or schools or preschool$ or pre school$ or schoolage$).ti,ab.

11 8 or 9 or 10

*Cancer identification*

12 exp Neoplasms/

13 (cancer$ or neoplas$ or oncolog$ or malignan$ or tumo?r$ or sarcoma$

or leukaemi$ or leukemi$ or chemotherap$).ti,ab.

14 12 or 13

*Consolidation*

15 11 and 14

16 7 and 15

*CDR Hedge*

17 (predict$ or clinical$ or outcome$ or risk$).mp.

*Final search*

18 16 and 17
