# Supplementary material for: Predicting infectious complications in neutropenic children and young people with cancer (IPD protocol)
Source: Syst Rev. 2012 Feb 9;1:8. doi: 10.1186/2046-4053-1-8 (PMC3351734; doi:10.1186/2046-4053-1-8)
Supplement: Additional file 3 — Data collection survey. [file 2046-4053-1-8-S3.DOCX]

#
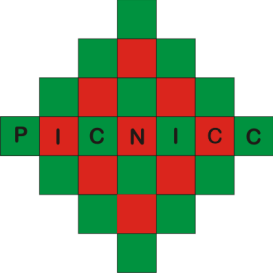
Appendix 3: Data collection survey.

Also available as web-form

**The PICNICC (Predicting Infectious Complications of Neutropenic sepsis In Children and young people with Cancer) Collaborative**

*Optimizing risk predictive strategies in febrile neutropenic episodes in children and young people undergoing treatment for malignant disease*

Checklist of potential data items for the PICNICC study

| Centre Name/Location |  |
| --- | --- |
| Principle clinician |  |
| Contact for data queries (if known) |  |

Please tick (√) each item to identify which information your dataset could or could not provide.

| **Item** | **Can provide** | **Cannot provide** | **Unsure** |
| --- | --- | --- | --- |
| ***Predictors*** | | | |
| Age |  |  |  |
| Underlying tumour type |  |  |  |
| Marrow involvement/remission status |  |  |  |
| Chemotherapy type and date of last cycle |  |  |  |
| Presence of central venous line |  |  |  |
| In-patient or out-patient at onset of episode |  |  |  |
| Maximum temperature |  |  |  |
| Respiratory rate (or compromise) |  |  |  |
| Circulatory (or compromise) |  |  |  |
| Severe mucositis |  |  |  |
| Global assessment of illness severity |  |  |  |
| Antibiotic therapy used |  |  |  |
| Haemoglobin |  |  |  |
| Platelet count |  |  |  |
| White cell count |  |  |  |
| Neutrophil count |  |  |  |
| Monocyte count |  |  |  |
| CRP |  |  |  |
| PCT |  |  |  |
| IL6 |  |  |  |
| IL8 |  |  |  |
| ***Outcome*** | | | |
| Death |  |  |  |
| Duration of intensive care admission |  |  |  |
| Need for moderate organ support (fluid bolus, oxygen) |  |  |  |
| Clinically documented infections |  |  |  |
| Microbiologically documented  infections |  |  |  |
| Duration of fever |  |  |  |
| Duration of admission |  |  |  |
| ***Other*** | | | |
| Date of episode of FNP |  |  |  |
| Age at episode of FNP |  |  |  |

Please return completed form to [crd-picnicc@york.ac.uk](mailto:crd-picnicc@york.ac.uk)
