# Supplementary material for: Predicting infectious complications in neutropenic children and young people with cancer (IPD protocol)
Source: Syst Rev. 2012 Feb 9;1:8. doi: 10.1186/2046-4053-1-8 (PMC3351734; doi:10.1186/2046-4053-1-8)
Supplement: Additional file 4 — Suggested coding structure. [file 2046-4053-1-8-S4.DOCX]

# Appendix 4: Suggested coding structure

**Age**

Actual age at start of episode, in months

999 = unknown

**Tumour type**

Diagnosis under treatment, coded as:

1. Acute lymphoblastic leukaemia
2. Acute myeloid leukaemia
3. Other leukaemia
4. Hodgkins lymphoma
5. Non-Hodgkins lymphoma
6. Low-grade brain tumour (I-II)
7. High-grade brain tumour (III-IV)
8. ‘High risk’ neuroblastoma
9. Other neuroblastoma
10. Retinoblastoma
11. Wilm’s tumour
12. Other renal tumour
13. Hepatoblastoma
14. Other liver tumor
15. Osteosarcoma
16. Ewing’s sarcoma
17. Rhabdomyosarcoma
18. Other sarcoma
19. Germ cell/gonadal neoplasm
20. Carcinoma/melanoma
21. LCH
22. Other Please provide separate details of any ‘other’ diagnoses

99 Unknown

**Relapsed/progressive disease**

Is this a relapsed/progressive malignancy:

0 = no

1 = yes

9 = unknown

**Marrow involvement**

Bone marrow involvement at diagnosis:

0 = no

1 = yes

9 = unknown

**Remission status**

In remission (leukaemia only) or on-treatment or post-treatment (solid tumours)

0 = no

1 = yes

9 = unknown

**Chemotherapy type**

Specify the most recent chemotherapy cycle (in words/by acronym or explicit numerical coding)

This will require a description of each chemotherapy protocol included from each study.

Specify ‘Unknown’ if unknown

**Time from last chemotherapy cycle**

Time (in days) since the start of most recent cycle of chemotherapy. For maintenance/prolonged chemotherapy courses, code as ‘ongoing’ even if temporarily discontinued.

0 = ongoing

1 ….k = time in days

999 = unknown

**Presence of central venous line**

0 = no

1 = fully implanted (e.g. Port-a-cath)

2 = external tunnelled (e.g. Hickman)

3 = non-tunnelled line (e.g. PICC line or Vascath)

4 = line present, type unknown

9 = unknown if line present or not

**In-patient or out-patient at onset of episode**

0 = in-patient

1 = out-patient

9 = unknown

**Maximum temperature**

Maximum recorded temperature at admission. May be parent-reported or clinician-measured.

To be recorded as an absolute value in ^0^C to one decimal place.

99.9 = unknown

**Respiratory assessment**

At initial assessment. To be recorded as an absolute value in breaths/min where given.

1 ….k = respiratory rate (breaths/min)

If data are only available on the presence/absence of respiratory compromise:

777 = no compromise

888 = compromised

999 = unknown

**Circulatory assessment - HR**

At initial assessment. To be recorded as an absolute value of heart rate in beats/min.

1 ….k = pulse rate (beats/min)

999 = unknown/not recorded

**Circulatory assessment – BP systolic**

At initial assessment. To be recorded as an absolute value mmHg.

1 ….k = systolic blood pressure (mmHg)

999 = unknown/not recorded

**Circulatory assessment – BP diastolic**

At initial assessment. To be recorded as an absolute value mmHg.

1 ….k = diastolic blood pressure (mmHg)

If data are only available on the presence/absence of circulatory compromise, code here:

777 = no compromise

888 = compromised

999 = unknown

**Mucositis**

At initial assessment. To be recorded as

0 = none

1 = mild

2 = severe

9 = unknown

**Global assessment of illness severity**

At initial assessment. To be recorded as

0 = well

1 = mildly unwell

2 = severely unwell

9 = unknown

(If an alternative study-specific system is available, please report and specify coding separately.)

**Initial antibiotic therapy**

The initial antibiotic therapy used should be reported. This can be done by specify the treatment used (in words/by acronym).

(Will require a description of each chemotherapy protocol included from each study)

Specify ‘Unknown’ if unknown

The PICNICC Secretariat will recode such information as below:

Initial antibiotic therapy coded as the PRODUCT of individual codes

0 = none

2 = oral antibiotics; quinilone

3 = oral antibiotics; penicillin

5 = oral antibiotics; macrolide

7 = IV antibiotics; cephalosporin

11 = IV antibiotics; carbapenem

13 = IV antibiotics; aminoglycoside

17 = IV antibiotics; piperacillin/tazobactam

19 = IV antibiotics; glycopeptide

(as a series of prime numbers, any number which is coded from them will be unique)

**Modification of antibiotic therapy**

Modification of antibiotic therapy required

0 = no

1 = yes

9 = unknown

**Haemoglobin**

At initial assessment. In mg/dL

9999 = unknown

**Platelet count**

At initial assessment. As count *10^9^

9999 = unknown

**White cell count**

At initial assessment. As count *10^6^

9999 = unknown

**Neutrophil count**

At initial assessment. As count *10^6^

9999 = unknown

**Monocyte count**

At initial assessment. As count *10^6^

9999 = unknown

**CRP**

At initial assessment. In mg/dL

9999 = unknown

**PCT**

At initial assessment. In mg/mL

9999 = unknown

**IL6**

At initial assessment. In pg/mL

9999 = unknown

**IL8**

At initial assessment. In pg/mL

9999 = unknown
